# Chronic pancreatitis: how does surgery step in?

**DOI:** 10.3389/fgstr.2026.1861029

**Published:** 2026-07-08

**Authors:** Luca Santagiuliana, Federico Gronchi, Tommaso Dall’Olio, Gabriella Lionetto, Matteo De Pastena, Giuseppe Malleo, Salvatore Paiella, Roberto Salvia

**Affiliations:** 1 Department of Pancreatic Surgery, University of Verona Hospital Trust, Verona, Italy; 2 Department of Surgery, Dentistry, Paediatrics and Gynecology, University of Verona, Verona, Italy; 3 Department of Engineering for Innovation Medicine (DIMI), University of Verona, Verona, Italy

**Keywords:** chronic pancreatitis, Frey procedure, pancreatic surgery, pancreaticoduodenectomy, total pancreatectomy with islet autotransplantation

## Abstract

Chronic pancreatitis (CP) is a progressive inflammatory disorder of the pancreas characterized by irreversible morphological changes that lead to chronic abdominal pain, exocrine and endocrine insufficiency, and a significant reduction in quality of life. Historically, surgical intervention was considered a last-line option, reserved for patients in whom conservative or minimally invasive therapies had failed. However, growing evidence suggests that early surgical management may provide superior outcomes in terms of pain control and quality of life. The surgical management of CP encompasses a spectrum of procedures tailored to disease morphology and symptom profile. These include drainage procedures for patients with a dilated pancreatic duct, formal pancreatic resections for localized inflammatory masses or in cases with a high suspicion of malignancy, and combined resection–drainage techniques when ductal dilatation coexists with an inflammatory mass in the pancreatic head. A multidisciplinary evaluation is recommended for all patients prior to intervention to ensure optimal patient selection and individualized treatment planning. This review provides a narrative synthesis of the current literature on surgical indications, timing, techniques, postoperative outcomes, and long-term results, offering a comprehensive overview of contemporary evidence and clinical decision-making in the surgical management of CP.

## 1 Introduction

**Figure 1:**
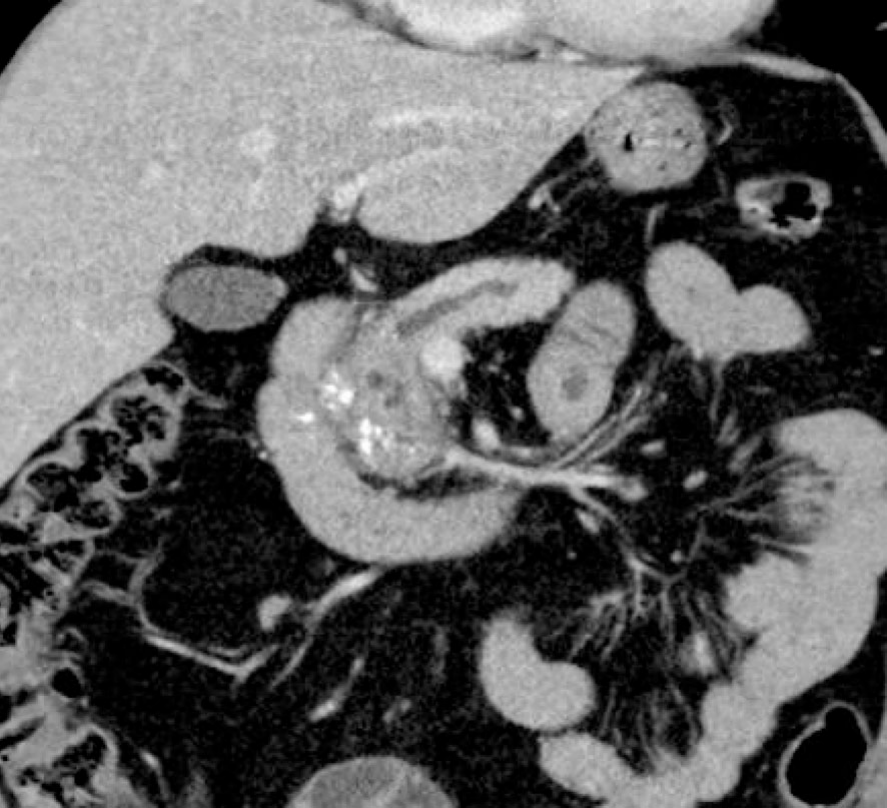
Pancreatic head calcifications with main duct dilation.

Chronic pancreatitis (CP) is defined as a pathological fibroinflammatory syndrome of the pancreas occurring in individuals with genetic, environmental, and/or other risk factors who develop persistent pathological responses to parenchymal injury or stress. This inflammatory process leads to fibrosis, ductal distortion, and calcifications, with progressive destruction of the pancreatic parenchyma. These changes result in several clinical consequences, including irreversible exocrine and endocrine insufficiency, as well as recurrent and severe abdominal pain that negatively affects patients’ quality of life. In addition, CP is associated with a range of local complications, including strictures of the pancreatic and/or biliary ducts, pseudocysts, pancreaticolithiasis, duodenal stenosis, and vascular complications (1) (**Figure 1**). Furthermore, CP is associated with an increased risk of pancreatic ductal adenocarcinoma, with a cumulative risk of approximately 1.8% at 10 years and 4% at 20 years of follow-up in sporadic CP. Lifetime risk is estimated at 5%–10% in patients with well-characterized genetic predispositions (2, 3).

### 1.1 Epidemiology, etiology, and diagnosis

The incidence of CP is 5–14 cases per 100,000 inhabitants, with a prevalence of approximately 30–50 per 100,000 individuals (4–9). The prevalence increases with age, and the median age at diagnosis ranges between 51 and 58 years (4, 9). Among etiological factors, alcohol and smoking are the leading causes of CP. Others include genetic mutations (in CFTR, SPINK1, PRSS1 genes), hypercalcemia, hypertriglyceridemia, certain medications, idiopathic mechanisms, and obstructive causes including main pancreatic duct obstruction (stenosis, calcification, masses) (10). Furthermore, approximately 10% of acute pancreatitis cases and up to 30% of recurrent acute pancreatitis cases may progress to CP (3). When clinical suspicion arises, pancreatic function should be assessed; the diagnosis of CP is subsequently established using cross-sectional imaging (CT or MRI with MRCP) and, when necessary, endoscopic ultrasound (EUS), which is considered the most sensitive imaging modality for the diagnosis of CP, particularly in cases where pancreatic cancer is suspected. Diagnosis relies on the identification of characteristic morphological features, including increased parenchymal density, glandular atrophy, calcifications, pseudocysts, and irregularities of the main pancreatic duct and its side branches. Histological evaluation is generally reserved for equivocal cases, particularly to exclude malignancy in patients presenting with an inflammatory mass (3, 11).

### 1.2 Pain as the leading indication for intervention

Pain is the most disabling and clinically dominant symptom in patients with CP, occurring in approximately 80%–90% of cases. Recurrent pancreatic inflammation, often associated with episodes of recurrent acute pancreatitis, results in episodic acute abdominal pain. Pain may arise from ductal hypertension due to ductal obstruction and inflammation, as well as from structural complications related to fibrosis, such as an inflammatory mass in the pancreatic head, pseudocysts, or pancreatic cancer. In addition, pain may have a neuropathic component, involving peripheral and/or central sensitization, which leads to the upregulation of proinflammatory and pronociceptive signaling pathways (12, 13). Even if pancreatic burn-out (improvement of pain symptoms) can occur, as described by Shalimar et al. in a study involving 288 patients showing 57% of patients were pain-free at the 5-year follow-up (14), this is inconsistent and unpredictable (11). As a consequence, pain remains the main indication for treatment of CP. The therapeutic management of CP has historically adhered to a step-up strategy, prioritizing medical and endoscopic interventions, with surgical treatment considered only in patients who fail to respond adequately to less invasive approaches (11, 15). A multimodal approach is central to CP management, primarily aiming at pain reduction and replacement of exocrine and endocrine functions. Treatment begins with dietary modifications, along with cessation of alcohol and smoking, as well as other lifestyle interventions. Analgesic therapy follows the principles of the WHO pain relief ladder, with the stepwise introduction of agents of increasing analgesic potency, titrated to achieve adequate pain control. Non-opioid analgesics are preferred as first-line therapy, with escalation to weak opioids (e.g., tramadol, codeine) and subsequently to strong opioids (e.g., morphine, oxycodone, fentanyl), depending on pain severity (16). Adjuvant analgesics are frequently used, including pregabalin, antidepressants, and anticonvulsants (2). When non-invasive treatments are insufficient, invasive procedures should be considered. In general, interventional treatment for pain in CP should be performed in experienced centers and reserved for patients with recurrent pain attacks unresponsive to non-opioid therapy, as well as for those with significant morphological changes. Endoscopy plays a central role in the management of CP, particularly in the treatment of pain and its complications, including pancreatic duct drainage, stent placement, and lithotripsy. Endoscopic ultrasound (EUS)-guided therapies have also been developed for targeted celiac plexus block or neurolysis to achieve pain relief. In clinical practice, endoscopic interventions may delay or, in some cases, obviate the need for surgical treatment. However, a substantial proportion of patients continue to experience persistent symptoms despite these measures (3). In this context, surgical treatment plays a crucial role, particularly in patients with refractory pain or advanced morphological disease. Increasing evidence suggests that early surgical intervention may provide superior long-term outcomes compared with delayed surgery, especially in terms of pain control and quality of life, underscoring the importance of timely multidisciplinary evaluation (17).

## 2 Methods

This narrative review summarizes current evidence on the surgical treatment of chronic pancreatitis. A literature search was conducted in PubMed and Embase to identify relevant articles published through January 2026. The search strategy included combinations of the following keywords: “chronic pancreatitis”, “surgical management”, “timing of surgery”, “early surgery”, “surgery versus endoscopy”, “endoscopic treatment”, “pain management”, “pancreatic drainage procedures”, “pancreatic resection”, “Frey procedure”, “Beger procedure”, “total pancreatectomy with islet autotransplantation”, and “surgical outcomes”. No formal predefined inclusion or exclusion criteria were applied. Literature identification was primarily guided by relevance to the topic, with preference given to international guidelines, systematic reviews, meta-analyses, randomized controlled trials, prospective and retrospective cohort studies, and landmark articles describing treatment of chronic pancreatitis. Non-English articles, case reports, conference abstracts and editorials were not considered. The literature was screened and selected by two authors based on relevance to the objectives of the review, with particular emphasis on surgical indications, operative techniques, postoperative outcomes, pain control, pancreatic function, and quality of life. In case of differing interpretations of the available evidence, consensus was reached through discussion among the authors. As this work was designed as a narrative review, no formal systematic review methodology or quantitative study quality assessment was performed. The findings should therefore be interpreted as a narrative synthesis of the available evidence.

## 3 Results

### 3.1 When and why to operate in chronic pancreatitis

Traditionally, a long period of medical pain management and multiple endoscopic interventions precede surgery. However, a substantial proportion of patients suffering from CP, ranging from 30%-75%, eventually necessitate surgical intervention, mainly because of persistent pain of mechanical complications related do morphological changes (15, 18, 19). In this context, several RCTs have demonstrated the superiority of early surgical intervention over less invasive endoscopic treatments in improving pain control and quality of life in patients with CP (**Table 1**), thereby challenging the traditional step-up approach to CP management (18, 20–23). However, these trials should be interpreted in the context of important methodological limitations, including relatively small sample sizes and clinical heterogeneity across studies, for example in patient selection, definitions of “early surgery, “ and outcome measures. Furthermore, the increasing availability of specialized pancreatic units, together with advances in pancreatic surgery and the associated reductions in morbidity and mortality, have strengthened the role of surgery in the management of selected patients with CP. The most common indication for surgery is intractable pain (3, 24). Although historically considered a last resort after failure of medical and endoscopic treatments, early surgery (within three years of symptom onset) may be associated with better outcomes, including higher rates of complete pain resolution and reduced narcotic use. Some studies also suggest potential improvements in quality of life and a possible association with lower rates of exocrine and endocrine insufficiency, although the evidence for these outcomes remains heterogeneous (24–27). A 2003 RCT on 72 patients by Dite et al. showed similar initial success between endoscopy and surgery, but at 5-year follow-up, complete pain relief was more frequent after surgery (34% vs 15%, P = 0.002), and more patients in the surgical group gained weight (47% vs 28%) (20). Similarly, in a 2007 RCT by Cahen et al. (39 patients), operative pancreaticojejunostomy resulted in lower Izbicki pain scores (P < 0.001) and better physical health (P = 0.003) over 24 months compared to endoscopic drainage. Complication rates, hospital stay, and pancreatic function were similar between groups, but endoscopic patients required more procedures (median 8 vs 3, P < 0.001). In the long term, initial surgery provided greater pain relief with fewer interventions, and nearly half of patients initially treated with endoscopy eventually required surgery (21, 22). More recently, the Dutch ESCAPE trial (88 patients with dilated main pancreatic duct and recent opioid use) showed that early surgery provided better pain control and improved quality of life compared to an endoscopy-first approach. In particular, during 18 months of follow-up, pain scores were lower in the surgery group (P = 0.02); however, this benefit was not consistently reflected across all clinical endpoints, as complete or partial pain relief at the end of follow-up did not differ significantly between groups (58% vs 39%, P = 0.10). Long-term follow-up of the same cohort (mean follow-up approximately 8 years) confirmed a sustained difference in pain outcomes, with lower Izbicki pain scores in the early surgery group (33% vs 51%, P = 0.03) and higher rates of complete pain relief (45% vs 20%, P = 0.04), as well as greater patient satisfaction and fewer reinterventions compared with the endoscopy-first strategy. Mortality, pancreatic function, and overall quality of life were similar, but early surgery required fewer interventions and was associated with a trend toward lower healthcare costs and higher patient satisfaction (18, 23, 28). Importantly, outcome measures differed across trials (e.g., Izbicki score vs numerical pain scales), further limiting direct comparison of effect sizes. Consistently, a 2014 review by Yang et al. reported that early surgery in CP may increase the likelihood of postoperative pain relief, may prevent pancreatic insufficiency, and reduces the need for repeated interventions, as nearly half of patients initially treated with endoscopy eventually required surgery (25). A multicenter European prospective study (ESCOPA) including 207 patients from 22 centers in 13 countries, showed significant pain relief and improved quality of life after surgical treatment for CP, with most patients relieved within six months and acceptable morbidity and mortality. Shorter symptom duration and absence of preoperative opioid use predicted complete pain relief, emphasizing the benefit of early surgery (29). Early surgery may therefore play both a therapeutic role, by addressing the underlying cause of pain, and a preventive role, by limiting ongoing pancreatic damage. Conversely, delayed surgery may increase the risk of opioid dependence and promote progression to neuropathic pain, which is notably more difficult to treat. However, although recent evidence supports early surgical intervention in CP, pancreatic surgery remains primarily focused on oncological indications. Despite the elevated pancreatic cancer risk linked to CP, surgical interventions for CP are commonly deferred in practice. This stems from pancreatic centers’ need to allocate limited resources to urgent oncological cases under established step-up treatment guidelines. Thus, although surgery plays an important role in selected patients, endoscopy still plays a central role in the management of CP, not only for pain relief but also for the treatment of local complications, with surgery generally reserved as a last resort when less invasive approaches fail. In this context, given the complexity of the disease, a multidisciplinary approach is always recommended. In addition to pain, common indications for surgical intervention include suspected neoplasia and local complications affecting adjacent organs, such as the common bile duct (CBD) or duodenum (24). CBD strictures causing recurrent acute cholangitis, obstructive jaundice, or persistent cholestasis are indications, primarily, for endoscopic stenting. In particular, the HaPanEU guidelines (11), suggest serial endoscopic approach may be considered in patients deemed suitable for repeated endoscopic retrograde cholangiopancreatography (ERCP) and who are at high surgical risk, present with portal hypertension, or have local abdominal conditions that contraindicate surgery. Resective surgery should also be considered in patients with an inflammatory mass in the head of the pancreas or in those with suspected neoplasia. Duodenal stenosis represents another indication for surgical intervention, particularly when conservative treatment has failed. In fact, duodenal subocclusion may be secondary to inflammatory edema and may resolve within 1–2 weeks. If resolution does not occur, an endoscopic or surgical bypass procedure (e.g., gastrojejunostomy) can be considered. Endoscopic gastro-duodenal stent placement is considered instead in patients who are not fit for surgery (30). In addition, surgery is considered in cases of pancreatic pseudocysts, even if guidelines recommend primarily endoscopic drainage (17). Pancreatic pseudocysts can be treated either by endoscopic (transmural or transpapillary) or surgical intervention, while percutaneous drainage is not recommended. While in the past, surgical procedures for treating pseudocysts were supposed to have higher success rates (31), in a RCT comparing endoscopic and surgical cystogastrostomy for the drainage of pancreatic pseudocysts, no recurrences were observed in the endoscopic group during follow-up, suggesting no clear superiority of the surgical approach (32). Furthermore, endoscopic treatment is associated with shorter hospital stays, better physical and mental health in patients, and lower costs (28). Other CP complications include pseudoaneurysms, whose treatment is primarily angiographic, while surgery represents a second-line and emergency treatment when embolization does not resolve the bleeding (33).

**Table 1:** RCTs comparing endoscopy and surgery in CP treatment.

Authors, year	Patients, n	Mean follow-up, months	Results
Dite (2003) (20)	72	60	Surgery: higher pain relief (86 vs. 61%), more increase in weight (47 vs. 29%)
Cahen (2007) (21)	39	24	Surgery: higher pain relief (75 vs. 32%), better physical quality of life; comparable morbidity
Cahen (2011) (22)	39	79	Surgery: higher pain relief (80% vs. 38%), fewer procedures (5% vs. 68%)
Issa (2020) (18)	88	18	Surgery: higher pain relief (58% vs. 39%); comparable mortality, pancreatic function, and quality of life
van Veldhuisen (2024) (23)	86	98	Surgery: higher complete pain relief (45% vs. 20%), fewer reinterventions (26% vs. 44%), more “satisfaction”

### 3.2 Surgical techniques: an overview

**Figure 2:**
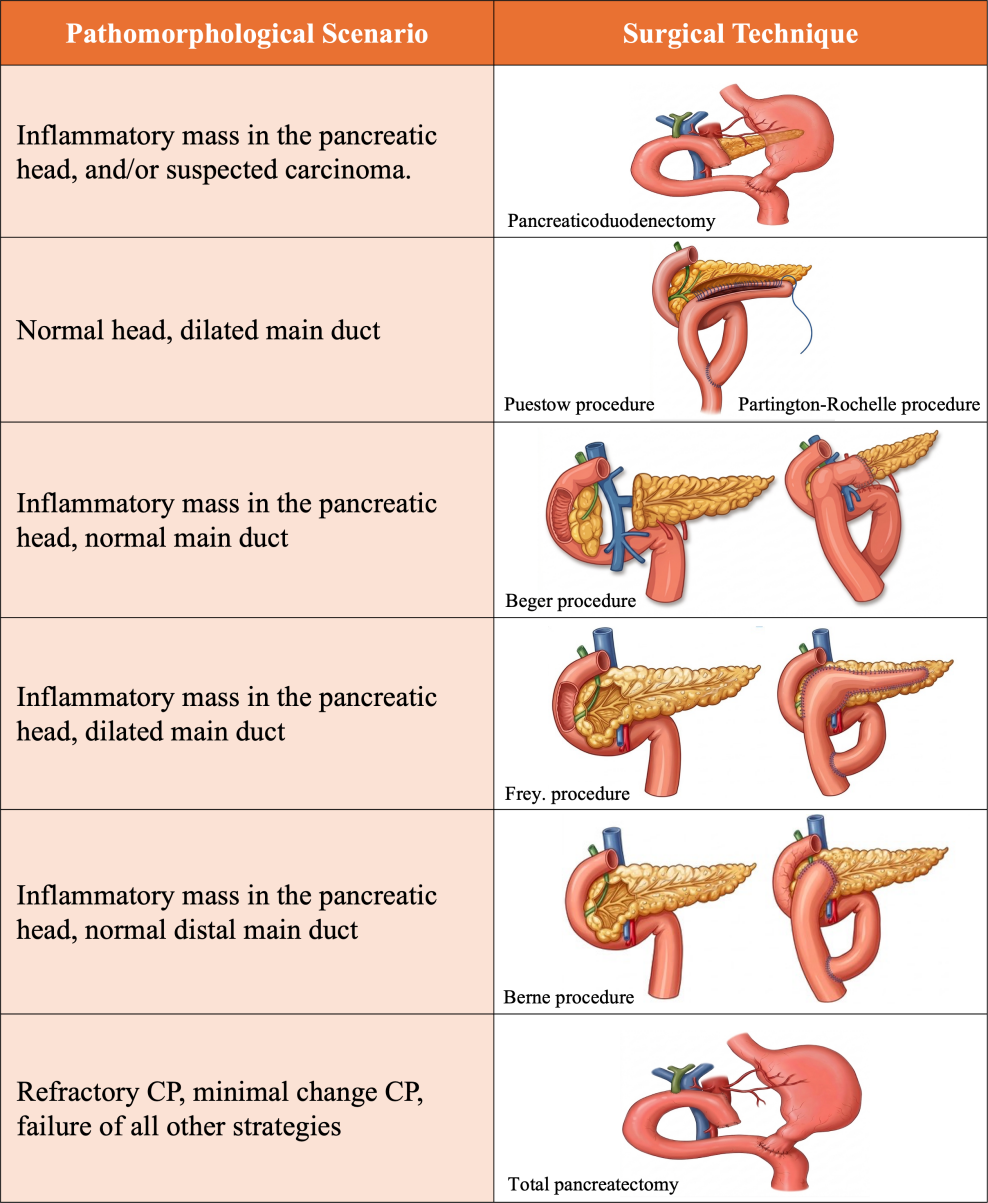
Strategies for the surgical treatment of chronic pancreatitis*.

The optimal surgical procedure should manage pain, preserve the maximum endocrine and exocrine function still present, resolve complications of adjacent structures, and restore quality of life. The surgical procedures adopted in CP management are traditionally divided into three categories: surgical drainage procedures, duodenum-preserving pancreatic head resections (DPPHRs), and formal pancreatic resections. The choice is based on the morphological features of the pancreas (e.g., an inflammatory mass in the head or tail, strictures/dilatation of the PD, and duct disruption) and on involvement of adjacent structures (e.g., duodenal or common bile duct stenosis and portal hypertension). Surgeon experience, practice, and level of comfort likely also affect operative choice (**Figure 2**). Traditionally, surgical interventions for CP have been performed using open approaches; however, minimally invasive techniques, particularly robotic-assisted methods, are increasingly being adopted in high-volume centers.

#### 3.2.1 Special challenging scenario: portal cavernomatosis

**Figure 3:**
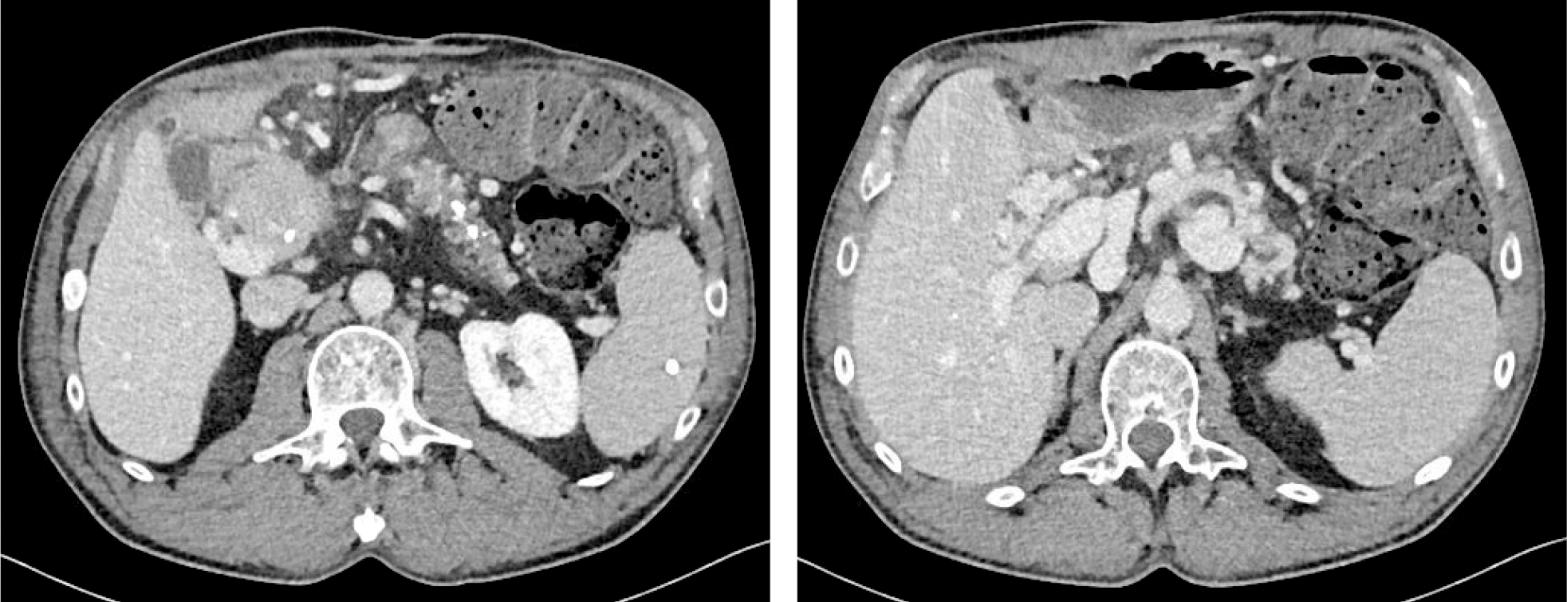
Chronic pancreatitis complicated by portal cavernomatosis.

Portal cavernomatosis represents a relevant anatomical and technical challenge that may significantly influence the choice of surgical approach in CP. Occurring in approximately 3%–12% of patients with CP, portal cavernoma develops as a consequence of chronic splanchnic venous thrombosis (**Figure 3**). Its development significantly increases operative risk, with morbidity rates reaching 88% compared with 35% in patients without vascular complications, and mortality of 10% versus 1.3%. However, it should not be considered an absolute contraindication to surgery. Despite more challenging surgical procedures, longer operative times, and greater transfusion requirements, operative intervention often remains the only definitive treatment option, and the substantial improvements in quality of life and pain relief justify it despite the increased perioperative risk (34–36). The choice of surgical procedure must be tailored to the presence of portal cavernoma. In patients with compression or occlusion of the portal vein system, which occurred in 12% of patients in the ChroPac trial, duodenum-preserving pancreatic head resection (DPPHR) should be the procedure of choice, since it avoids dissection of the pancreas from the portal and superior mesenteric veins, reducing the risk of major bleeding and other intraoperative complications (37).

#### 3.2.2 Drainage procedures

Drainage procedures are needed when a dilated main duct exists, i.e., a main duct diameter of 5 mm or more in the pancreatic body, and when this condition does not coexist with an inflammatory focus in the pancreatic head. In 1954, first Zollinger and then DuVal described internal retrograde drainage of the pancreas by pancreaticojejunostomy, performing a distal pancreatectomy with splenectomy (38). Improving this procedure, Puestow and Gillesby introduced a lateral (longitudinal) opening of the pancreatic duct, including removal of the pancreatic tail and spleen (39). Subsequently, Partington and Rochelle presented a modification of the Puestow-Gillesby approach in 1960 (40). In this procedure, the PD is opened along the anterior surface of the pancreas, from the tail to somewhat to the right of the mesenteric vessels. The dilated Wirsung duct is easily palpated at the isthmus/body due to its firmness amid the hardened gland. If difficult, fine-needle aspiration or intraoperative ultrasound can be used. Ductal stones are then eventually removed with forceps and/or high-pressure irrigation. The duct is then anastomosed to a 60–70 cm Roux-en-Y jejunal limb, proceeding in a tail-to-head fashion. The procedure is associated with low morbidity and mortality rates (about 1%). Immediate and lasting pain relief is reported in 80% (range 42–100%) of patients, with a follow-up of 62 (range 15–110) months (41, 42). Because the majority of the obstructed ducts in the pancreatic head are left undrained, extended lateral pancreaticojejunostomy is recommended, in which the pancreatic duct is opened to within 1–2 cm of the splenic hilum and within 1 cm of the duodenum, to decompress the entire pancreatic ductal system, including the ducts of Wirsung and Santorini (43). In the case of extended lateral pancreaticojejunostomy, the right gastroepiploic and pancreaticoduodenal branches of the gastroduodenal artery need to be ligated. In patients with isolated distal pancreatic duct obstruction, an extended lateral pancreaticojejunostomy is preferred over distal pancreatectomy. Distal pancreatectomy unnecessarily increases the risk of developing endocrine insufficiency and should therefore be avoided in patients with CP (44).

#### 3.2.3 Duodenum preserving pancreatic head resections

In patients with CP who present with an inflammatory mass in the pancreatic head, with or without dilation of the main pancreatic duct and/or side branches in the head (and body/tail) region, the treatment of choice consists of combined drainage–resection procedures, including the Frey, Beger, Berne, and V-shaped excision procedures. The Frey procedure consists of a pancreaticojejunostomy combined with coring out of the pancreatic head, leaving a narrow rim of pancreatic tissue along the duodenum, the posterior aspect of the head, and adjacent to the portal and mesenteric veins. The procedure begins with a lateral pancreaticojejunostomy. As the dissection approaches the pancreatic head, the right gastroepiploic vessels and the anterior pancreaticoduodenal branch of the gastroduodenal artery must be ligated to allow resection of the pancreatic head. During this step, an adequate amount of pancreatic parenchyma must be preserved, especially on the duodenal side, in order to maintain its vascularization (45). The Beger procedure, first described in 1980, consists of a subtotal resection of the pancreatic head with preservation of the duodenum by a rim of pancreatic parenchyma along the inner duodenal wall, which contains the duodenal arterial blood supply. Two anastomoses are created between a Roux-en-Y jejunal limb, the proximal remaining portion of the pancreatic head, and the distal pancreatic body (46). The Berne procedure involves coring out of the pancreatic head and subsequent pancreaticojejunostomy using a Roux-en-Y limb, but without the extended lateral pancreaticojejunostomy that is created in the Frey procedure (47). A V-shaped excision, i.e., the Hamburg procedure, is applied in CP without a dilated main duct. To preserve as much pancreatic tissue as possible and to lower the risk of postoperative exocrine and endocrine pancreatic dysfunction, a longitudinal V-shaped excision of the ventral pancreas is performed, which aims not only to drain the main duct but also the secondary ductal branches (48, 49).

#### 3.2.4 Formal pancreatic resections

This group includes pancreaticoduodenectomy, left pancreatectomy, and total pancreatectomy. Pancreaticoduodenectomy (PD) consists of removal of the pancreatic head, duodenum, distal bile duct, gallbladder, and proximal jejunum (10–15 cm); the pylorus-preserving variant (Longmire–Traverso) spares the stomach (50), while Whipple/Kausch procedure includes antrectomy (51). Reconstruction is performed via pancreaticojejunostomy, hepaticojejunostomy, and gastrojejunostomy. This procedure is used in CP presenting with an inflammatory mass in the pancreatic head with or without a dilated pancreatic duct, especially when a malignancy cannot be ruled out. These procedures provide long-term pain relief in 75–95% of the patients. The major disadvantage of a pancreaticoduodenectomy for CP is the removal of the surrounding non-diseased organs, such as the duodenum and the entire pancreatic head, leading to significantly reduced pancreatic exocrine and endocrine functions (52). Furthermore, PD represents the most suitable surgical option for patients with groove pancreatitis (44). Left pancreatectomy involves resection of pancreatic tail/body, often with splenectomy for vascular control. During the 1960s and 1970s, a left pancreatectomy became the most performed operation for pain relief in CP. Later left pancreatectomy became less used because of the high incidence of endocrine and exocrine insufficiencies the concomitant development of other less aggressive surgical procedures for the treatment of CP (52). Total pancreatectomy consists in complete gland excision with duodenectomy, often splenectomy; variants preserve pylorus if feasible; alternatively, antrectomy is performed. Reconstruction requires hepaticojejunostomy and gastrojejunostomy. Total pancreatectomy alone can lead to so-called brittle diabetes. This scenario can be avoided by combining total pancreatectomy with islet autotransplantation (TPIAT), first described in 1970s, in which pancreatic islet cells are isolated and infused into the liver via the portal vein so that they will engraft in the liver parenchyma. For patients with refractory CP despite having had operative treatment or whose pancreatitis is hereditary, a TPIAT can be the last resort for treatment. A systematic review and meta-analysis by Kempeneers et al., reported showed a 63% opioid-free rate, a 30% insulin-free rate, and a statistically significant improved quality of life at 1 year after TPIAT (53). The timing of operation for these conditions, however, is also important because an early procedure will result in a greater yield of islets, and a late procedure potentially leads to less pain relief (54). Nonetheless, precise optimal timing of this surgical procedure remains undefined in the literature.

### 3.3 Many procedures – which one to choose?

Several RCTs have been performed comparing duodenum preserving pancreatic head resections (DPPHR) with PD, showing overall no significant differences (37, 55–59). In 2008, a systematic review and meta-analysis by Diener et al. comparing DPPHR with PD found no significant differences in postoperative pain relief, overall morbidity, incidence of postoperative pancreatic fistula, or operative time. However, the Frey procedure was associated with a significantly shorter operative time, reduced rates of delayed gastric emptying, shorter hospital stay, and a lower need for perioperative blood transfusion compared with PD. Furthermore, patients undergoing DPPHR showed improved quality of life and greater postoperative weight gain, although this was accompanied by a higher rate of exocrine pancreatic insufficiency compared with the PD group (60). Similarly, a systematic review and meta-analysis of RCTs by Zhao et al. including 385 patients and comparing DPPHR vs PD found no significant differences in postoperative mortality, pain relief, long-term exocrine or endocrine insufficiency. However, DPPHR showed several advantages, including shorter operative time, fewer transfusions, reduced hospital stay, lower postoperative morbidity, greater weight gain, improved quality of life and better occupational rehabilitation (59). The multicente, double-blind ChroPac trial demonstrated that, after 24 months of follow-up, DPPHR and partial PD produced no meaningful differences in quality of life (including pain assessment), suggesting that any early functional advantages of duodenal preservation may attenuate with time and/or be influenced by postoperative management and disease progression (37). Similarly, in a prospective randomized study with 85 patients randomized to urdergo either pylorus-preserving (PPPD) or DPPHR, Keck et al. reported shorter operative time with DPPHR (360 vs 435 minutes) but otherwise comparable perioperative safety (overall complications 33% vs 30%; pancreatic leakage 10% vs 5%; reoperation 2% vs 2%; mortality 0% in both arms) and comparable longer-term outcomes after >5 years (pain control 67% vs 67%; endocrine impairment 45% vs 44%; exocrine insufficiency 76% vs 61%; and no difference in quality of life) (58). In a large single-center contemporary series of 338 pancreatic head resections (2007–2023), major morbidity was similar after PD vs DPPHR (23% vs 21%), but PD had longer operative time and higher blood loss; 30-day mortality was low overall (1.2%), occurring only after PD (1.6% vs 0%) (61). In a nationwide cohort of morphology-based surgery (381 patients), drainage operations were associated with lower major complications (12% vs 24% vs 26%; *P* = 0.012), lower clinically relevant postoperative pancreatic fistula (POPF grade B/C) (0% vs 3% vs 22%; *P* = 0.038), fewer reoperations (%vs 16% vs 12%; *P* = 0.006), and 90-day mortality of 0% compared with head resections or PD (vs 3% and 2%; *P* = 0.139). Functionally, the same cohort also demonstrated lower new-onset endocrine insufficiency after drainage (14% vs 21% vs 43%; *P* < 0.001), while new-onset exocrine insufficiency did not differ significantly between groups (45% vs 41% vs 51%; *P* = 0.571), underscoring that drainage can preserve pancreatic reserve, while reducing early morbidity (62). Overall, across randomized trials, DPPHR and PD achieve broadly equivalent long-term symptom control, supporting procedure selection primarily on morphology and the need to exclude malignancy, rather than expectations of differential pain efficacy. In clinical practice, the final decision is also frequently influenced by surgeon experience and institutional expertise. Among DPPHR, a systematic review and meta-analysis comparing the Frey with the Beger procedures showed no difference in post-operative pain, mortality, morbidity, exocrine insufficiency and endocrine insufficiency (59, 63). A network meta-analysis by Ratnayake et al., including 597 patients, revealed that the Beger procedure is associated with the highest pain relief, whereas the Frey procedure is favorable in terms of postoperative quality of life, pancreatic fistula rate, and exocrine insufficiency, thus representing the best overall compromise (64).

### 3.4 Surgical approach: open vs minimally invasive

The advent of minimally invasive surgery has been associated with several potential benefits, including reduced postoperative pain, shorter hospital stay, and a lower incidence of wound-related complications (65). However, the current evidence base is largely derived from retrospective and observational studies, which are inherently subject to selection bias, heterogeneity in disease severity, and differences in institutional expertise. Within this context, recent retrospective studies have suggested that laparoscopic approaches are safe and effective in the surgical management of CP, providing outcomes comparable to open surgery in terms of pain relief and overall complication rates, while being associated with longer operative times but reduced intraoperative blood loss (65–67). Nevertheless, given the technical complexity of these procedures, laparoscopic surgery for CP should be performed in specialized, high-volume pancreatic centers. In particular, the Miami guidelines on minimally invasive pancreatic surgery recommend that pancreatic resections should be performed in highly selected patients and only in centers meeting specific implementation requirements, including structured training, completion of the learning curve, and adequate annual case volume, in order to ensure patient safety and optimal outcomes (68). Indeed, robotic surgery has been increasingly proposed as a means to overcome some of the technical limitations of conventional laparoscopy, particularly for complex pancreatic procedures. However, robust comparative evidence remains limited. The robotic approach for lateral pancreaticojejunostomy in CP is under active investigation through the PANACOTTA RCT (NCT07109180), which compares robot-assisted versus open procedures in terms of postoperative recovery quality and safety. This multicenter study targets patients with symptomatic CP and dilated pancreatic ducts (≥ 5 mm), with a non-enlarged pancreatic head < 40 mm. At present, robotic approaches should therefore be considered investigational, with their potential advantages likely confined to selected high-volume centers with advanced minimally invasive expertise. Overall, while minimally invasive techniques appear promising, open surgery remains the reference standard, particularly in complex or advanced disease, until more definitive randomized evidence becomes available.

## 4 Conclusions

**Figure 4:**
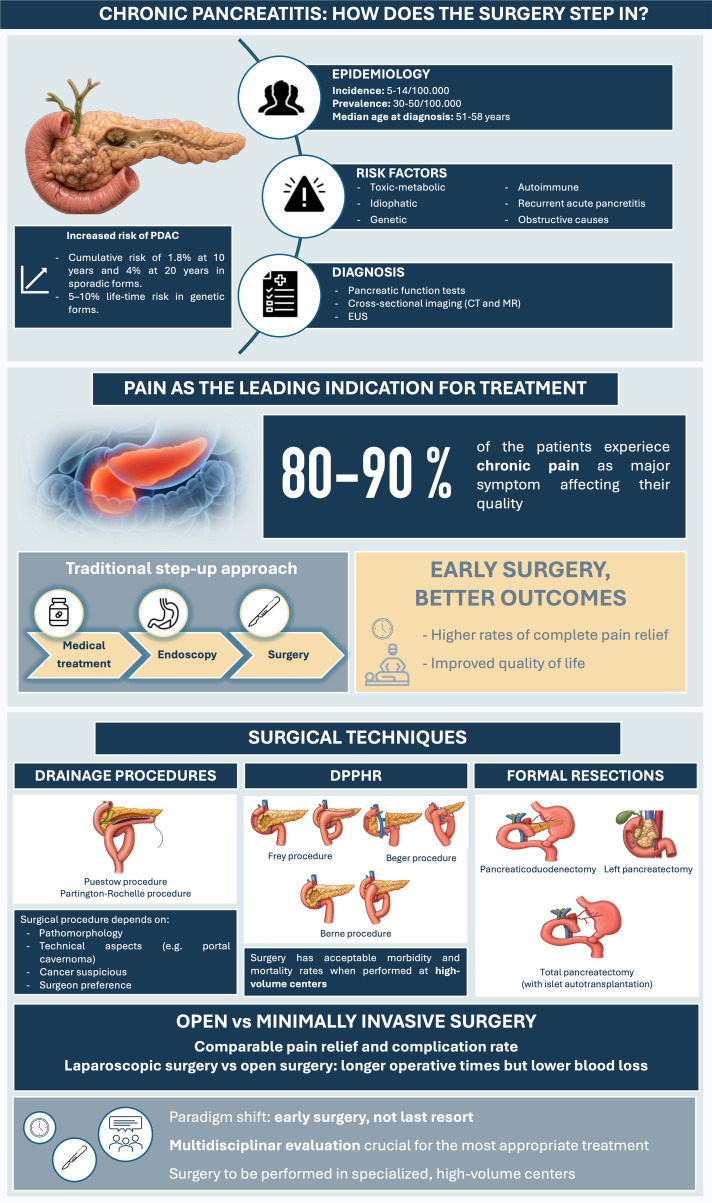
Infographic*.

In conclusion, surgical management of CP represents a key component in the treatment of this complex and multifactorial disease, particularly in patients with refractory pain and local complications not manageable through conservative or endoscopic approaches. Surgical techniques range from drainage procedures to resection and combined interventions. They must be carefully selected based on the gland’s morphological features, disease distribution, and the patient’s clinical condition. Early surgery appears to be associated with superior pain control, reduced disease progression, and better preservation of pancreatic function, whereas delaying surgery may allow irreversible changes, including central sensitization and advanced fibrosis, ultimately limiting the benefits of operative treatment. A multidisciplinary evaluation of patients with CP is crucial to define the most appropriate treatment, which is often multimodal (**Figure 4**).
